# The Role of Attachment Insecurity in the Intergenerational Transmission of Violence

**DOI:** 10.1007/s40653-025-00766-2

**Published:** 2025-09-17

**Authors:** Matthew Gregg Saxsma, Rachel C. Garthe

**Affiliations:** 1 Department of Psychology, University of Illinois at Urbana-Champaign, 603 E. Daniel St., Champaign, IL 61820 United States of America; 2 School of Social Work, University of Illinois at Urbana-Champaign, Champaign, IL United States of America

**Keywords:** Intimate partner violence, Perpetration, Adverse childhood experiences, Attachment insecurity, Gender

## Abstract

**Purpose:**

Intimate partner violence (IPV) is a pervasive problem associated with a plethora of negative outcomes, including depression, post-traumatic stress disorder, and chronic illness. Prior research has identified adverse childhood experiences (ACEs) as an important antecedent to IPV perpetration, and various mechanisms have since been examined to explain this association, including attachment. The purpose of this research is to investigate the mediating role of insecure adult romantic attachment in the relationship between ACEs and IPV perpetration, while also examining group differences among men and women.

**Methods:**

The current study included a cross-sectional sample of 395 young adults between the ages of 18 and 24 (M_age_ = 19.1; 64% cisgender female; 54% White). We fit structural equation models to investigate the relationship between ACEs and IPV perpetration and the mediating role of attachment insecurity.

**Results:**

In women, part of the relationship between ACEs and IPV perpetration is mediated by attachment anxiety. In men, attachment anxiety plays a mediating role despite no total effect of ACEs on IPV perpetration. Attachment avoidance did not mediate the association between ACEs and IPV in men or women.

**Conclusions:**

Attachment anxiety may play a role in the intergenerational transmission of violence. These findings can be used by clinicians and practitioners to prevent the development of an anxious attachment orientation or target anxious attachment in adults.

**Supplementary Information:**

The online version contains supplementary material available at 10.1007/s40653-025-00766-2.

Although romantic relationships can confer many perceived benefits, they can also generate negative experiences, including intimate partner violence perpetration (IPV-P), which encompasses a wide variety of physical, sexual, and psychologically abusive behaviors in the context of dating relationships (CDC, 2021). While the general population experiences IPV at high rates (CDC, 2021), studies have found college students to be at a particularly high risk of IPV victimization and perpetration. Anywhere from 26 to 45% of college students report physically assaulting a romantic partner while in college (Brennan et al., 2021; Nabors, 2010; Nabors & Jasinski, 2009; Straus, 2004). Experiencing IPV is associated with negative health outcomes, including depressive symptoms, post-traumatic stress disorder, chronic disease, lower academic efficacy, and lower scholastic achievement (Banyard et al., 2020; Campbell, 2002; Coker et al., 2002). Given the severe negative impact of IPV, researchers have sought to inform prevention efforts for this form of violence perpetration.

## Childhood Adversity and the Intergenerational Transmission of Violence

An important finding of IPV research is that witnessing violence or being a victim of violence in one’s family of origin can be an antecedent of violent behavior in adulthood (Widom & Wilson, 2015). This has been called the “intergenerational transmission of violence” (ITV; Haselschwerdt et al., 2019). The ITV has been supported in a variety of empirical studies (e.g., Low et al., 2017; Stith et al., 2000; Whitfield et al., 2003). However, one criticism of the existing ITV literature has been the limiting focus on individual types of violence, such as physical violence (Haselschwerdt et al., 2019). Studies have examined childhood physical abuse, sexual abuse, and violence exposure individually, but rarely are they examined together (Jennings et al., 2015; Narayan et al., 2017). Capturing multiple forms of violence and adversity simultaneously may help to broaden our understanding of what kinds of violence are transmitted across generations.

For instance, one way to capture more diversity is by examining adverse childhood experiences (ACEs; Felitti et al., 1998). ACEs include physical, emotional, and sexual abuse, neglect, and household adversities (e.g., witnessing intrafamilial violence, having a caregiver with substance use problems, or forced separation from a caregiver) that occur from ages 0–18 years (CDC, 2022). More research is needed to examine ACEs in relation to adulthood violence to better understand the link between exposure to childhood adversity and violence in adulthood. A handful of studies have found that ACEs are associated with greater levels of IPV victimization (e.g., Thulin et al., 2021) and perpetration (e.g., Fonseka et al., 2015; Gilchrist et al., 2017; Lee et al., 2022) in adulthood. For example, Fonseka and colleagues (2015) found that exposure to four or more ACEs was associated with IPV perpetration among a sample of Sri Lankan men. Although the link between ACEs and IPV-P has been examined, more research is needed to examine the underlying mechanisms in this relationship. In addition, research is needed to consider differences by gender, as a handful of studies have focused on the relationship between ACEs and IPV-P with male samples (e.g., Fonseka et al., 2015; Gilchrist et al., 2017; Lee et al., 2022). Prior research has begun to investigate attachment bonds in the ITV, but their role remains unclear. The current study aims to address this gap in the literature by examining insecure (i.e., anxious and avoidant) attachment styles as potential mediators of the relationship between ACEs and IPV-P, while also considering gender differences, among a sample of college students.

## The Role of Attachment

Attachment bonds were first studied by Bowlby (1973) in order to understand the relationship between an infant and a caregiving figure. An integral part of attachment bonds is what he called “internal working models” (Bowlby, 1982; Simpson et al., 2020). These are the mental representations of attachment related experiences, including thoughts and feelings related to attachment figures (Bowlby, 1982; Simpson et al., 2020). Building off this work, Hazan and Shaver (1987) sought to conceptualize adult romantic love as an attachment bond, consisting of working models of romantic partners. Brennan and colleagues (1998) then found that two primary factors underlie thoughts and feelings toward romantic partners: attachment anxiety and avoidance. Attachment anxiety refers to feelings of uncertainty about a partner’s responsiveness and fears of abandonment. Attachment avoidance entails being uncomfortable with intimacy, distancing oneself from others, and an emphasis on self-reliance in romantic relationships (Mikulincer et al., 2003). Being low in both avoidance and anxiety constitutes a “secure” attachment style and being high in either or both dimensions constitutes an “insecure” attachment style. A child who experiences neglectful and abusive caregiving will be more likely to develop a negative internal working model of their caregiver, in which they view the caregiver as unreliable, absent, or rejecting (Mikulincer et al., 2003). This model then guides the child in navigating their attachment bonds with their caregivers and romantic partners later in life (Fraley et al., 2011, 2013). For example, attachment insecurity in romantic relationships is associated with relationship satisfaction (Candel & Turliuc , 2019). In short, attachment experiences have implications for relationship experiences throughout the lifespan.

### Attachment and IPV

Attachment is of interest in the ITV because being in a romantic relationship with an insecure attachment style may facilitate interpersonal violence. Several scholars have put forth ideas for how attachment insecurity might lead to intimate partner violence. For example, Mayseless (1991) proposed that people high in attachment anxiety may use violence to communicate their desire for physical and psychological proximity in response to cues of rejection or abandonment from their romantic partners. People high in attachment avoidance may use violence as a way to maintain independence and psychological distance from their partners (Mayseless, 1991). Others have suggested that people with an insecure attachment style may have a harder time with differentiating their own mental states and actions as well as the actions and mental states of others, as a result of not developing these skills in attachment relationships throughout development (Fonagy, 2003; Fossati et al., 2009). Thus, violence may be more likely in cases where an understanding of mental states that cause a romantic partners attachment-relevant actions may inhibit a violent response.

There is ample empirical support for an association between attachment insecurity and IPV perpetration. Researchers have shown that both adult attachment anxiety and avoidance are correlated with physical, sexual, and psychological forms of IPV-P (Sommer et al., 2017; Spencer et al., 2021; Velotti et al., 2022). Additionally, the association between attachment insecurity and violence perpetration has been observed in clinical and community populations (Ogilvie et al., 2014).

### ACEs, Attachment, and IPV-P

To date, only a handful of studies have examined the association between ACEs and IPV-P as mediated by attachment styles, and these studies have produced inconclusive results. Gay and colleagues (2013) found that only early maladaptive schemas, but not attachment styles, significantly mediated the relationship between childhood emotional abuse and physical IPV-P. However, early maladaptive schemas, which are memories, thoughts, and feelings about oneself and one’s relationship with others, are clearly conceptually related to attachment styles and may have introduced collinearity into their model, resulting in non-significant findings of the mediational role of attachment (Gay et al., 2013; Thimm, 2010; Young et al., 2003). Second, a study by Lee and colleagues (2014) found that attachment anxiety was a significant mediator in women in the relationship between physical abuse and physical dating violence perpetration, but this was not found in men. A third study, by Sutton et al. (2014), used structural equation modeling to analyze the relationship between hostile parenting and dating violence perpetration. They found that avoidant attachment and anxious attachment, along with the belief that relationship disagreements are threatening, fully mediated this relationship in men and partially mediated the relationship for women. However, none of their tested models simultaneously included anxious and avoidant attachment. Finally, Godbout et al. (2009) and Godbout et al. (2017) analyzed models featuring attachment insecurity as mediators between early exposure to violence and IPV-P. Godbout et al. (2009) found that attachment anxiety and avoidance were significant mediators in their model. Godbout et al. (2017) found that attachment anxiety partially mediated the relationships between exposure to family of origin violence and relationship violence perpetration, and that attachment avoidance was not related to either early exposure to family violence or perpetrated relationship violence.

In sum, prior research on the relationship between childhood maltreatment, attachment, and IPV-P has shown several inconsistencies. There is disagreement about the role of attachment avoidance in transmitting violence across generations (Godbout et al., 2009, 2017; Lee et al., 2014; Sutton et al., 2014). Additionally, sex and gender differences among these relationships are not fully understood. One study failed to examine men (Gay et al., 2013), and several found conflicting evidence for gender invariance of the theoretical model (Godbout et al., 2009, 2017; Lee et al., 2014; Sutton et al., 2014). Furthermore, prior studies have each only focused on a few types of childhood maltreatment, namely physical, emotional, and psychological abuse (Gay et al., 2013; Godbout et al., 2017; Lee et al., 2014). More research that sheds light on the role of attachment bonds in these relationships and includes broad aspects of an adverse childhood could be beneficial.

### The Current Study

Support for the ITV has been evidenced by researchers finding significant associations between specific adverse childhood experiences and cumulative ACEs in relation to adult IPV-P. However, significant gaps exist in the literature that the current study seeks to address. First, we will examine the overall association between ACEs and IPV-P. Based on prior research, we hypothesize there to be a positive association between ACEs and IPV-P. Second, we will examine the mediating role of anxious and avoidant attachment styles. Attachment theory and previous empirical findings suggest that both anxious and avoidant attachment styles will mediate this relationship (Godbout et al., 2017; Lee et al., 2014; Sutton et al., 2014). Third, we will examine sex differences in the model. Given the large discrepancies that previous researchers have found, this research aim is exploratory.

## Methods

### Participants

The sample used in this study included 395 students from a large midwestern university in the United States who indicated that they had dated or been in a relationship in the past. The sample was 63.8% female and racially diverse (55% White, 26% Asian/Asian American, 15% Black/African American, 13% Mexican/Mexican American; participants could select multiple options for race and ethnicity). Participants’ ages ranged from 18 to 24 (*M* = 19.1, *SD* = 0.95). Demographic information can be found in Table 1.

**Table 1:** Sample characteristics (*N* = 395)

Characteristic	*N*	%
Sex		
Male	142	35.9
Female	252	63.8
Not Sure	1	0.3
Race and Ethnicity		
Asian or Asian American	102	25.8
Black or African American	59	14.9
Cuban	3	0.8
Mexican, Mexican American, Chicano/a/x	51	12.9
Middle Eastern or Northern African	6	1.5
Native American, American Indian, or Alaskan Native	4	1.0
Native Hawaiian or Other Pacific	1	0.3
Puerto Rican	13	3.3
White	215	54.4
Another Race or Ethnicity	11	2.8
Age		*M (SD)*
		19.1 (0.95)

Note: M = mean, SD = standard deviation

### Procedure

This study obtained IRB approval. The survey used to obtain the data was designed on REDCap and distributed to 5000 (2500 first year and 2500 s year) students at a large, midwestern, public university in April of 2021. In total, 720 surveys were completed. All participants provided online consent. However, 159 respondents submitted more than one survey or failed the attention check questions; these surveys were removed from the final sample, leaving 561 complete surveys. Of those, 395 indicated that they had dated or were currently dating. The data used in this research cannot be shared due to privacy restrictions.

### Measures

***ACEs***. Experiences with childhood (before the age of 18) adversity were measured with ten dichotomous items (Felitti et al., 1998). These items included forms of childhood abuse, neglect, and household dysfunction (e.g., “Did a parent or other adult in your household often: Swear at you, insult you, put you down, or humiliate you? OR act in a way that made you afraid that you might be physically hurt?”). Participants answered Yes = 1 or No = 0 to each of the ten items. All 10 items were summed to create an overall ACEs score. ACEs questionnaires show criterion validity in prior research (Giano et al., 2021; Lee et al., 2020; Oláh et al., 2023).

#### Attachment Insecurity

The Experiences in Close Relationships-Short Form (Wei et al., 2007) was used to measure insecure attachment. The ECR-Short Form is a 12-item measure that captures attachment on the anxious and avoidant dimensions. Both anxiety and avoidance were answered on a 5-point scale ranging from 1 = Strongly Disagree to 5 = Strongly Agree. Items 4, 5, 6 and 10 were reverse scored. Both measures were adequately reliable (α_anxiety_ = 0.725; α_avoidance_ = 0.813). The study that documented the development of the ECR-Short Form found evidence for the criterion validity of the measure (Wei et al., 2007).

Attachment was modeled as a latent factor in this study. Confirmatory factor analysis (CFA) affirmed the avoidant and anxious factor structure. The reverse coded items yielded small (< 0.400) factor loadings and were removed from all descriptive and SEM analyses (Matsunaga, 2010). This left five items loading on Attachment Anxiety (loadings ranging from λ = 0.494 to λ = 0.785) and three items loading on Attachment Avoidance (loadings ranging from λ = 0.756 to λ = 0.896). Results of the CFA are listed in Table 2. Finally, the modification indices revealed correlating the residuals of item 8 and item 9 would improve the model fit. This correlation is justifiable because both of these items explicitly pertain to “closeness” with partners.

**Table 2:** Confirmatory factor analysis of the experiences in close relationship short form

ECR-Short Form Item	λ	SE	λ^*^	SE^*^
Factor 1: Avoidance				
**1. want to get close but pulling back**	0.887	0.054	0.756	0.027
**2. nervous when partners get close**	1.065	0.052	0.896	0.022
**3. avoid getting close**	0.827	0.047	0.800	0.025
4. discuss problems				
5. turn to in times of need				
6. turn to for comfort and reassurance				
Factor 2: Anxiety				
**7. reassurance I am loved**	0.613	0.065	0.525	0.047
**8. like partners to be close**	0.551	0.061	0.515	0.049
**9. desire to be close scares**	0.586	0.062	0.534	0.048
10. do not worry about abandonment				
**11. get frustrated when unavailable**	0.572	0.065	0.494	0.048
**12. partner doesn’t care as much**	0.956	0.068	0.785	0.042

Note: χ2(18) = 49.238, *p* = .000, CFI = 0.967, TLI = 0.949, SRMR = 0.058, and RMSEA = 0.067, with 90% confidence interval (CI) [0.045, 0.090]. Non-bolded items were dropped from the final model. λ * = standardized factor loading, λ = unstandardized factor loading, SE* = standardized standard error, SE = standard error. Items are paraphrased. See Wei et al. (2007) for the full items

#### IPV-P

The Conflict in Adolescent Relationships Inventory Short Form (CADRI-S; Fernández-González et al., 2012) was used to measure IPV experiences. It is a 20-item survey, with 10 items for victimization (e.g., “My partner kicked, hit, or punched me”) and 10 for perpetration (e.g., “I kicked, hit, or punched my partner”). Only the perpetration items were examined for the current study. Items were answered on a scale of 0 = Never, 1 = Once, 2 = 2 or more times and then dichotomously coded 0 = Never or 1 = At least once. IPV-P consisted of five subscales: Physical, Threats, Sexual, Psychological, and Verbal. The overall measure of IPV-P to be used was created by summing all perpetration items. The original CADRI and the short form we are using were originally developed on samples of adolescents with mean ages 14–18 (Fernández-González et al., 2012; Wolfe et al., 2001). The CADRI has also shown to be a reliable measure in young adult samples (ages 19–25; López-Barranco et al., 2022). The original CADRI demonstrates adequate test-retest reliability, interrater reliability, and convergent validity (Wolfe et al., 2001).

### Data Analyses

Means and standard deviations were calculated using complete cases, and correlations were calculated for all variables of interest using pairwise complete cases. We used the lavaan package and the cfa() and sem() functions in RStudio to fit Structural Equation Models (SEM; R Core Team, 2021; Rosseel, 2012). The first SEM examined the associations between ACEs and IPV-P, with attachment anxiety and avoidance as mediating variables. Most people report low levels of IPV-P, so the distribution of IPV is positively skewed (i.e., nonnormal). Thus, the multivariate normality assumption of traditional maximum likelihood (ML) estimation in SEM is heavily violated and standard ML may not be tenable. Instead, we used maximum likelihood with robust standard errors (MLR) to protect against issues arising from nonnormality (Yuan & Bentler, 2000).

To assess the fit of this SEM, a series of fit indices were reviewed. The chi-square test statistic is used to indicate good fit, with higher values indicating worse fit. However, it is influenced by sample size, with larger samples likely to call for model rejection. Other fit indices such as the comparative fit index (CFI; Bentler, 1990), Tucker-Lewis index (TLI; Tucker & Lewis, 1973), Root Mean Squared Error of Approximation (RMSEA; Steiger & Lind, 1980), and Standard Root Mean Square Residual (SRMR; Jöreskog & Sörbom, 1981) are used to supplement the chi-square goodness of fit test. The CFI and TLI range from 0 to 1, with larger values indicating better fit (Kline, 2015). The RMSEA and SRMR have a lower bound at 0, with lower values indicating better fit (Kline, 2015). MLR yields a robust version of the chi-square goodness of fit test statistic (Satorra & Bentler, 2001). As such, fit indices dependent on this statistic, such as the CFI, TLI, and RMSEA, are all robust versions (Walker & Smith, 2017).

Next, we examined differences between men and women in this model by conducting multiple group analysis. This analysis involves sequentially constraining and relaxing each pathway, examining changes in Akaike Information Criterion (AIC; Akaike, 1998) and Bayesian Information Criterion (BIC; Schwarz, 1978), and conducting Chi-Square difference tests to determine improvement in model fit. All structural pathways were constrained and relaxed to explore potential sex differences. The direct and indirect pathway effects will be estimated using bootstrapping methods (Preacher & Hayes, 2004). All path coefficient estimates reported are fully standardized.

## Results

### Descriptive Statistics and Bivariate Correlations

Descriptive statistics and bivariate correlations can be found in Table 3. On average, participants scored moderately high on attachment anxiety (*M* = 2.919, *SD* = 0.791) and attachment avoidance (*M* = 2.338, *SD* = 1.001). Women scored slightly higher on attachment anxiety (*M* = 2.990, *SD* = 0.759) and lower on attachment avoidance (*M* = 2.313, *SD* = 0.967) than men (attachment anxiety: *M* = 2.783, *SD* = 0.830; attachment avoidance: *M* = 2.386, *SD* = 1.063).

Correlations were computed in R and are presented in Table 3 (R Core Team, 2021). All variables were significantly and positively correlated with each other, except attachment avoidance and IPV-P, which had a non-statistically significant correlation. Among women, all variables were positively correlated. In men, only attachment anxiety was correlated with avoidant attachment and ACEs.

A total of 26.49% (*n* = 102) of the participants reported perpetrating at least one act of IPV in their lifetime. The various forms of IPV were reported at unequal rates (Physical 7.27%, Threats 4.43%, Sexual 3.65%, Psychological 4.18%, Verbal 22.05%). Men and women differed in reported rates of perpetration as well, with 23.78% of men and 43.43% of women committing at least one act of violence.

The majority (63.2%; *n* = 249) of participants in our sample experienced at least one ACE, and 19.5% (*n* = 77) reported experiencing four or more ACEs, which is in line with previous research (Merrick et al., 2019). Emotional abuse was the most common ACE (37.95%), followed by physical abuse (24.81%), emotional neglect (23.08%), sexual abuse (6.85%), and physical neglect (5.12%). Women in this sample experienced higher levels of every ACE compared to men. All reported percentages of ACEs were calculated with the whole sample.

**Table 3:** Descriptive statistics and correlations

	Women			Men						
Variable	*n*	*M*	*SD*	*n*	*M*	*SD*	1	2	3	4
1. Attachment Anxiety	252	2.990	0.759	141	2.783	0.830	—	0.184^*^	0.215^*^	0.127
2. Attachment Avoidance	252	2.313	0.967	141	2.386	1.063	0.192^**^	—	0.116	-0.123
3. ACEs	252	2.131	2.283	141	1.284	1.774	0.195^**^	0.152^*^	—	0.049
4. IPV-P	248	0.657	1.279	136	0.390	1.005	0.177^**^	0.161^*^	0.314^***^	—

Note: ACEs = adverse childhood experiences, IPV-*P* = intimate partner violence perpetration, M = mean, SD = standard deviation. * *p* < .05; ** *p* < .01; *** *p* < .001. Men’s correlations are on top of the diagonal

### Overall SEM

The first model showed acceptable fit, χ^2^_(36)_ = 82.074, *p* < .001, CFI = 0.942, TLI = 0.912, SRMR = 0.060, and RMSEA = 0.061 with 90% confidence interval (CI) [0.044, 0.078]. ACEs were directly (β = 0.203, *p* = .006) associated with IPV-P and indirectly associated with IPV-P via anxious attachment (β = 0.047, *p* = .040). The pathway from attachment avoidance to IPV-P was not statistically significant (β = 0.034, *p* = .582). This model explained 9.4% of the variance in IPV-P. All results for the overall SEM are shown in Table 4.

**Table 4:** Pathway coefficients of the overall structural equation model (*N* = 345)

	Anxiety						Avoidance					IPV-*P*				
	B	SE	β		SE	*R* ^2^	B	SE	Β	SE	*R* ^2^	B	SE	β	SE	*R* ^2^
						0.08					0.04					0.09
Age	-0.04	0.07	-0.04		0.06		0.01	0.06	0.00	0.05		0.02	0.05	0.03	0.03	
ACEs	0.14^***^	0.03	0.29^***^		0.06		0.09^**^	0.03	0.20^**^	0.06		0.11^**^	0.04	0.20^**^	0.07	
Anxiety												0.19^*^	0.08	0.16^*^	0.07	
Avoidance												0.04	0.07	0.03	0.06	
ACEs →Anxiety →IPV-P	0.03^*^	0.01	0.05^*^		0.02											
				ACEs →Avoidance →IPV-P			0.00	0.01	0.01	0.01						

Note: χ^2^_(36)_ = 82.074, *p* < .001, CFI = 0.942, TLI = 0.912, SRMR = 0.060, and RMSEA = 0.061 with 90% confidence interval (CI) [0.044, 0.078]. ACEs = adverse childhood experiences, IPV-*P* = intimate partner violence perpetration. * *p* < .05; ** *p* < .01; *** *p* < .001. *B* = unstandardized estimate, *β* = standardized estimate

### Multiple-Group Analysis

To construct the group model, all pathways were sequentially constrained and relaxed. This left three equality constraints on the pathways from ACEs to attachment anxiety, attachment anxiety to IPV-P, and ACEs to attachment avoidance. The pathways from attachment avoidance to IPV-P and the direct pathway from ACEs to IPV-P were allowed to vary between women and men. The group model showed acceptable fit, χ^2^_(78)_ = 157.588, *p* < .001, CFI = 0.910, TLI = 0.873, SRMR = 0.071, and RMSEA = 0.077 with 90% confidence interval (CI) [0.060, 0.094]. The results from the group model are shown in Table 5 (for women) and 6 (for men). Among women, all individual pathways were significant in the expected positive direction, except for avoidance to IPV-P (β = 0.118, *p* = .083). ACEs were directly (β = 0.244, *p* = .003) related to IPV-P and indirectly (β = 0.048, *p* = .029) related to IPV-P via attachment anxiety. We did not detect a significant indirect effect of ACEs on IPV-P via attachment avoidance (β = 0.026, *p* = .119). These results suggest mediation by attachment anxiety in the relationship between ACEs and IPV-P of women. This model explained approximately 15.2% of the variance in IPV-P among women.

**Table 5:** Pathway coefficients of the women’s structural equation model (*N* = 229)

	Anxiety						Avoidance					IPV-*P*				
	B	SE	β		SE	*R* ^2^	B	SE	β	SE	*R* ^2^	B	SE	β	SE	*R* ^2^
						0.08					0.05					0.15
Age	-0.06	0.08	-0.04		0.06		-0.00	0.06	0.00	0.05		0.05	0.04	0.03	0.03	
ACEs	0.12^***^	0.03	0.27^***^		0.07		0.10^**^	0.03	0.22^**^	0.07		0.14^**^	0.05	0.24^**^	0.07	
Anxiety												0.22^**^	0.08	0.18^**^	0.07	
Avoidance												0.15	0.09	0.12	0.06	
ACEs →Anxiety →IPV-P	0.03^*^	0.01	0.05^*^		0.02											
				ACEs →Avoidance →IPV-P			0.02	0.01	0.03	0.02						

Note: χ^2^_(78)_ = 157.588, *p* < .001, CFI = 0.910, TLI = 0.873, SRMR = 0.071, and RMSEA = 0.077 with 90% confidence interval (CI) [0.060, 0.094]. ACEs = adverse childhood experiences, IPV-*P* = intimate partner violence perpetration. * *p* < .05; ** *p* < .01; *** *p* < .001. *B* = unstandardized estimate, *β* = standardized estimate

**Table 6:** Pathway coefficients of the men’s structural equation model (*N* = 115)

	Anxiety						Avoidance					IPV-*P*				
	B	SE	β		SE	*R* ^2^	B	SE	β	SE	*R* ^2^	B	SE	β	SE	*R* ^2^
						0.05					0.03					0.06
Age	-0.06	0.08	0.03		0.15		-0.00	0.06	0.03	0.11		0.05	0.04	0.03	0.06	
ACEs	0.12^***^	0.03	0.22^***^		0.06		0.10^**^	0.03	0.18^**^	0.06		-0.03	0.05	-0.05	0.09	
Anxiety												0.22^**^	0.08	0.25^**^	0.08	
Avoidance												-0.13	0.12	-0.14	0.13	
ACEs →Anxiety →IPV-P	0.03^*^	0.01	0.05^*^		0.02											
				ACEs →Avoidance →IPV-P			-0.01	0.01	-0.03	0.03						

Note: χ^2^_(78)_ = 157.588, *p* < .001, CFI = 0.910, TLI = 0.873, SRMR = 0.071, and RMSEA = 0.077 with 90% confidence interval (CI) [0.060, 0.094]. ACEs = adverse childhood experiences, IPV-*P* = intimate partner violence perpetration. * *p* < .05; ** *p* < .01; *** *p* < .001. *B* = unstandardized estimate, *β* = standardized estimate

**Fig. 1:**
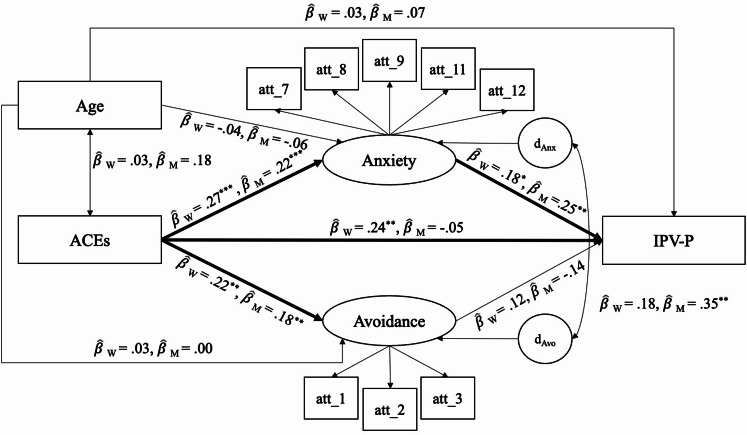
Structural equation model of the relationship between ACEs and IPV-P and the mediating role of attachment insecurity. IPV-P is regressed on Attachment Anxiety, Attachment Avoidance, Age, and ACEs. Anxiety and Avoidance are regressed on Age and ACEs. Age and ACEs are exogenous variables in the model. Attachment Avoidance is a latent variable measured with three indicators and Attachment Anxiety is a latent variable measured with five indicators. Note: The Structural Equation Model of the relationship between ACEs and IPV-P and the mediating role of attachment insecurity. This model controlled for age (*N* = 344). All associations between covariates and study variables are listed in Tables 4 and 5, and 6. χ^2^_(78)_ = 157.588, *p* < .001, CFI = 0.910, TLI = 0.873, SRMR = 0.071, and RMSEA = 0.077 with 90% confidence interval (CI) [0.060, 0.094]. Standardized coefficients are presented for both women (β_W_) and men (β_M_). Paths with at least one significant coefficient are bolded

Among men, all pathways were significant in the expected positive direction, except for the direct effect from ACEs to IPV-P (β = -0.053, *p* = .558) and from attachment avoidance to IPV-P (β = -0.143, *p* = .288). ACEs were indirectly associated with IPV-P via attachment anxiety (β = 0.048, *p* = .029). Additionally, we did not detect a significant indirect effect of ACEs on IPV-P via attachment avoidance for men (β = -0.032, *p* = .301). The significant indirect effect of ACEs on IPV-P via attachment anxiety and the lack of a significant direct effect suggests mediation by attachment anxiety in the relationship between ACEs and IPV-P among men. This model explains approximately 5.7% of the variance in IPV-P of men. Please see Fig. 1 for the SEM diagram, with differences specified for men and women.

## Discussion

The current study investigated the role of insecure adult romantic attachment styles in the intergenerational transmission of violence (ITV). Additionally, we investigated sex differences in these relationships. It was hypothesized that there would be a positive relationship between ACEs and IPV-P, and that this relationship would be mediated by insecure attachment styles. Overall, SEM techniques revealed that attachment anxiety mediated the relationship between ACEs and IPV-P. However, we also found evidence for sex differences in these relationships. These findings provide further support for the special role attachment anxiety may play in the ITV, and they contribute to the literature on the way attachment may play a different role in the ITV among men and women.

These results are consistent with prior literature that found childhood adversity to be positively associated with IPV-P via attachment anxiety (Godbout et al., 2009, 2017; Lee et al., 2014; Sutton et al., 2014). This may be because anxiously attached individuals are hypervigilant to threats of abandonment and may respond to these with violence (Mayseless, 1991). The current study did not find a relationship between avoidance and IPV-P. This may be because the attachment system of avoidant individuals is characterized by deactivation and distancing, meaning avoidant individuals do not seek as much proximity and intimacy in the face of attachment threats (Mikulincer et al., 2003; Shaver & Mikulincer, 2002). This may preclude the desire to act violently. However, the relationship between attachment avoidance and IPV-P requires further examination. It may be that there is no association between avoidance and IPV-P broadly, but there may be a relationship between avoidance and specific forms of violence. For example, sexual violence may result from failed attempts at establishing intimacy by avoidant individuals (Birnbaum et al., 2006).

The current study further contributed to our understanding of IPV by examining group differences among women and men. First, we found the direct relationship between ACEs and IPV-P was significant among women, but not men. The model estimates of the female group are consistent with prior research and attachment theory (e.g., Lee et al., 2014; Mikulincer et al., 2003). However, the lack of a direct effect between ACEs and IPV-P in men was unexpected (Li et al., 2020). This may be due to countervailing effects of other mediators (Zhao et al., 2010) or inaccurate reports of childhood maltreatment or IPV by males (Archer, 1999; Hilton et al., 2003; Fleming et al., 2015). Finally, the relatively small sample of men (*N* = 115) may not have provided enough statistical power to detect this relationship. Previous research that found evidence for the association between ACEs and IPV in men utilized larger samples (e.g., *N* = 315; Godbout et al., 2009).

Second, the group analyses indicated that the relationship between attachment avoidance and IPV-P is different between men and women. However, both the male and female group estimates for this pathway were not statistically significant in a final model with necessary constraints. That is to say, the difference between them was statistically significant, but they did not show a statistically significant difference from zero individually. The lack of a relationship between avoidance and IPV-P is in line with previous literature (e.g., Lee et al., 2014), although this may be an artifact of a small sample size of men. Additionally, one study did find evidence for the positive relationship between avoidance and IPV-P in both men and women (Godbout et al., 2009). The inconsistency of these findings warrants further study of the link between attachment avoidance and IPV-P, specifically with respect to sex differences.

This study provided evidence for sex differences in the relationships between ACEs, attachment insecurity, and IPV-P. This study contributes to our understanding of the etiology of intimate partner violence by directly comparing women and men, including both attachment anxiety and avoidance in the same model, and modeling attachment as a latent factor. To our knowledge, only one study has done similar analyses, and they found no sex differences (Godbout et al., 2009). The current study did find differences between women and men, but future research is needed to clarify these relationships.

The current study addressed several weaknesses of prior research. First, this study asked participants about household dysfunction, such as separation from a caregiver and having a parent with substance abuse issues, in addition to forms of abuse and neglect. These additional childhood experiences may play an important role in how a child forms attachment bonds. For example, several of the ACE items relate to caregiver availability. Having a caregiver absent due to incarceration or having divorced parents may prevent children from perceiving caregivers as reliable, secure bases (D’Rozario & Pilkington, 2022; Eddy & Poehlmann-Tynan, 2019). These early experiences with caregivers could then lay the groundwork for how individuals approach relationships with romantic partners in adulthood (Hazan & Shaver, 1987). Additionally, this study further strengthened this literature by modeling attachment as a latent factor. By doing this, we have controlled for measurement error where past studies did not.

### Limitations and Directions for Future Research

The current study is not without limitations. Although the total sample size was moderate (*N = 395)*, there were only 229 women and 115 men included in the group analyses. Future research should aim to use larger samples. This will not only allow for more accurate estimates, but larger samples will also allow more covariates to be included in the analyses. The present study was only able to control for age in the overall and group analyses. Perhaps accounting for more covariate effects will provide a more accurate depiction of the relationships between ACEs, attachment, and IPV-P. Also, while the current study examined binary sex differences, future work may wish to consider differences across diverse gender identities.

Another consideration with respect to sample size is the measurement model we used in our analyses. During the original measurement model construction, Wei and colleagues (2007) arrived at a bifactor model that included attachment anxiety and avoidance as latent factors, as well as two latent factors to account for bias in responding to positively and negatively worded items. We were not able to fit this measurement structure and conduct group invariance testing, likely due to the small sample size of men. Instead, we opted for a simpler structure based only on the latent factors of attachment anxiety and avoidance. This allowed us to conduct the multiple groups analysis. Larger sample sizes in future research will enable researchers to implement more complex measurement structures.

Additionally, the current study employed a cross-sectional design that only examined individuals. Future research should incorporate complex longitudinal designs that utilize the relationship dyad as the unit of analysis. There is evidence that accounting for a partner’s attachment orientation may affect the relationships between actor attachment and IPV-P (Sommer et al., 2017). However, it is also not enough to examine discrete acts of abuse, neglect, and violence. Measures should begin to capture the severity, chronicity, and context in which these acts occur (Haselschwerdt et al., 2019; Holden, 2003). Much of the prior literature has focused on a few types of abuse and/or violence perpetration, which limits generalizability of the role attachment plays in ITV (Godbout et al., 2017; Lee et al., 2014; Spencer et al., 2021). Longitudinal studies could also investigate whether psychopathology has an effect above and beyond attachment insecurity. For example, a generalized anxiety disorder diagnosis might explain part of the relationship between attachment anxiety and IPV-P, given that attachment insecurity is a risk factor for the development of psychopathology (Cassidy et al., 2009).

The current study utilized the ACEs Original Scale that captured ten kinds of abuse, maltreatment, and childhood adversity and an IPV measure that captured five kinds of violence (Felitti et al., 1998; Fernández-González et al., 2012). This widens the scope of violent experiences, but future research should separate these forms of violence to understand the contribution to attachment of specific acts of abuse and the effect of attachment on various forms of IPV. In fact, there is evidence that childhood abuse and maltreatment involving a strong emotional component (e.g., emotional abuse, emotional neglect, and psychological abuse) may be especially important in fostering an insecure attachment style (Dutton et al., 1994; Dutton & White, 2012; Riggs & Kaminski, 2010; Struck et al., 2020). Effectively separating these forms of violence will also require a large sample in order to deal with high levels of collinearity between them (Midi et al., 2010). Additionally, the ACEs scale used in the current research was created in a western context. Researchers interested in the effects of ACEs on health outcomes in adulthood should be mindful that racial, ethnic, and cultural differences may moderate the effects of childhood adversity (Yang et al., 2025). Results from studies of childhood adversity should not be improperly generalized across these contexts (Alhowaymel et al., 2021).

Although the CADRI-S contains items about violence perpetration, it only provides information about specific acts. It should be noted that violence can occur in response to violence; some people might indicate they have perpetrated an act of violence only because they were victimized first. The CADRI-S does not provide this context, so some respondents may have been victims of IPV before using violence themselves.

Finally, the overall model explained 9.4% of the variance in IPV-P and the indirect effects of attachment anxiety and avoidance accounted for approximately 20.6% of the shared variation between ACEs and IPV-P. This indicates that there are many more mechanisms undergirding the ITV. These may include emotion regulation, attitudes, self-control, and substance use (Cascardi & Jouriles, 2018; Eckhardt & Massa, 2020). Researchers may want to include these as mediators in the future.

### Clinical Implications

Despite these limitations, the current study found that having attachment anxiety may play a special role in transmitting violence across generations. This finding reveals potential strategies for intervention. There are evidence-based therapies, such as Trauma-focused Cognitive Behavioral Therapy (TF-CBT; Cohen et al., 2012), that are suited to deal with the complex and intergenerational nature of trauma, especially in treating children with extremely insecure attachments (e.g., disorganized; Pleines, 2019). Interventions and therapies employed once the child has already grown up with an insecure attachment style, such as Cognitive Behavioral Therapy/Psychodynamic Therapy (CBT/PT; Lawson, 2010; Lawson et al., 2012), can focus on attenuating the characteristics of anxious attachment (Zalaznik et al., 2017). Given that this is a sample of college students, universities and campus settings may wish to employ more trauma-informed treatment approaches, relationship workshops, violence prevention programs, and attachment-based therapies. By increasing the variety of supports that campuses can offer, perhaps universities can play a role in reducing the amount of violence that is perpetrated.

## Conclusions

The present study provides more insight into the role of anxious attachment in the intergenerational transmission of violence. Furthermore, this role may be different for men and women, as evidenced by the differences in mediation. These findings can be used by clinicians and practitioners to target attachment anxiety and prevent IPV-P. More broadly, the present study can be used by anyone curious to understand how a history of childhood maltreatment can be associated with violence in romantic relationships years later. Future research should continue increasing our understanding of the role of insecure attachment styles, so that we may curtail the intergenerational transmission of violence.

## Supplementary Information

Below is the link to the electronic supplementary material.

Supplementary Material 1

## Data Availability

The data cannot be shared due to privacy restrictions.
