# Supplementary material for: The Role of Attachment Insecurity in the Intergenerational Transmission of Violence
Source: J Child Adolesc Trauma. 2025 Sep 17;19(1):67–79. doi: 10.1007/s40653-025-00766-2 (PMC13004794; doi:10.1007/s40653-025-00766-2)
Supplement: Supplementary file 1 — Supplementary Material 1 [file 40653_2025_766_MOESM1_ESM.docx]

June 10, 2025

To whom it may concern,

Thank you for the opportunity to resubmit our manuscript titled “The Role of Attachment Insecurity in the Intergenerational Transmission of Violence” for possible publication in the *Journal of Child and Adolescent Trauma*.

The study makes several notable contributions to the line of research described in the paper. First, we used the Adverse Childhood Experiences Scale to measure events of abuse and maltreatment in childhood. This scale includes experiences that are frequently measured in the literature (e.g., physical abuse), as well as events less frequently measured (e.g., parent abusing substances). Second, this study further addressed the role of romantic attachment avoidance in the link between childhood adversity and perpetrating violence in romantic relationships. This was a point of inconsistency among prior findings. Third, we explored sex differences in our model. Much of previous research has not been able to simultaneously include men and women in their statistical models.

Based on our findings, our paper includes a rich discussion about the implications of this work. For example, we discuss how clinical interventions may use this research. Specifically, clinicians may want to focus efforts on preventing people from forming insecure attachment bonds with romantic partners.

In light of the reviewer comments, we made several revisions to the manuscript, and we thank you for allowing us to strengthen this work. We revised the discussion section to include important considerations brought up by reviewers, including caveats about the measures we used, and future directions regarding the role that psychopathology plays in intimate partner violence.

Again, thank you for the opportunity to submit our work for possible publication in the *Journal of Child and Adolescent Trauma*. We believe this paper is an excellent fit for the journal, has numerous contributions to the interventional transmission of violence research, and it has strong clinical implications.

Thank you.
